# Programmed cell death 4 (PDCD4) suppresses metastastic potential of human hepatocellular carcinoma cells

**DOI:** 10.1186/1756-9966-28-71

**Published:** 2009-05-29

**Authors:** Shuhong Zhang, Jianfeng Li, Ying Jiang, Yijun Xu, Chengyong Qin

**Affiliations:** 1 Department of Gastroenterology, Shandong Provincial Hospital Affiliated to Shandong University，324 Jingwu Weiqi Road, Jinan 250021, PR China; 2 Department of Gastroenterology, Jinan Central Hospital Affiliated to Shandong University，105 Jiefang Road, Jinan 250013, PR China; 3 Central Laboratory, Shandong Provincial Hospital Affiliated to Shandong University，324 Jingwu Weiqi Road, Jinan 250021, PR China

## Abstract

**Background:**

Hepatocellular carcinoma (HCC) is a lethal malignancy with high rate of metastasis and poor prognosis. There are no effective managements to block metastasis of HCC. Programmed cell death 4 (PDCD4) is found to be a tumor transformation suppressor. Among investigations on effects of PDCD4, little is about the metastatic potentials of HCC cells. This study was to investigate the role of PDCD4 on metastatic potential of human HCC cells.

**Methods:**

We examined the expression of PDCD4 in three HCC cell lines with different metastatic potentials, MHCC-97H (high metastatic potential), MHCC-97L (low metastatic potential) and Hep3B (no metastatic potential). A plasmid encoding PDCD4 gene was constructed and then transfected into HCC cells with the lowest PDCD4 expression level. Effects of PDCD4 on cell proliferation, cell apoptosis, gene expression of metastasis tumor antigen 1 (MTA1) and in vitro migration and invasion capacity were assessed after transfection.

**Results:**

Our results showed that the expression level of PDCD4 was inversely correlated to the metastatic potential of HCC cells. After transfection with the PDCD4 gene, HCC cell proliferation rate was significantly decreased, cell apoptosis rate was significantly increased, the expression of MTA1 gene, HCC cell migration and Matrigel invasion were also remarkably inhibited.

**Conclusion:**

PDCD4 expression is inversely correlated to the metastatic potential of HCC cells. PDCD4 can effectively suppress the metastatic potential of HCC cells.

## Introduction

Hepatocellular carcinoma (HCC) is a frequent and lethal malignancy with high rate of metastasis, especially in some regions of Africa and Asia [1]. It ranks the sixth most common cancer of men and 11th one of women worldwide. There were more than half a million deaths per year. The number of new HCC cases occurring each year is almost equivalent to the number of deaths [2,3]. Since HCC is clinically silent at early stage, most HCC patients (> 80%) are presented with advanced or unresectable disease. Without treatment, the 5-year survival rate of HCC is less than 5%. To those with resected disease, the recurrence rate can be as high as 50% at 2 years and the 5 year survival rate is only 25–39%. Despite of the advances in treatment, the prognosis of HCC remains very poor due to the frequent presence of recurrence and the high rate of metastasis [3-5].

The programmed cell death 4 (PDCD4) was found to be an inhibitor of neoplastic transformation. It was first found to be highly expressed during apoptosis, but the role of PDCD4 in programmed cell death was not clear. A comparative study on cells with different transformation response to tumor promoters revealed that PDCD4 was expressed more than ten folds higher in promotion-sensitive cells than in promotion-resistant cells. In less progressed mouse keratinocytes, higher level of PDCD4 was expressed [6]. Later investigations demonstrated that loss of PDCD4 expression was associated with tumor progression in carcinomas of the lung, colon, prostate, and breast [7]. The inhibition of PDCD4 on transformation is achieved through down-regulation of the JNK signal transduction pathway which is essential for cell migration. Decrease of JNK activity then leads to inhibition of cell migration [8,9].

The metastasis tumor antigen 1 (MTA1) was originally identified by differential expression in rat mammary adenocarcinoma metastatic cells [10]. The expression of the MTA1 gene was found to be positively correlated with metastatic potential of some human cell lines and tissues, such as the breast, prostate, colon and pancreas [11-13].

More and more researches on the effects of PDCD4 have been performed, among which little is about the relation of PDCD4 to metastatic potentials of HCC cells. Based on the previous studies, we hypothesized that PDCD4 might also play a role on the inhibition of HCC metastasis. To testify this hypothesis, we first examined the expressions of PDCD4 in three human HCC cell lines with different metastasis potentials, then we transfected a plasmid encoding the PDCD4 gene into HCC cells with lowest PDCD4 expression level and further investigated the effects of PDCD4 on the gene expression of MTA1 and migration and invasion of HCC cells.

## Methods

### Cell lines and cell culture

Three human HCC cell lines, MHCC-97H (high metastatic potential), MHCC-97L (low metastatic potential), Hep3B(no metastatic potential) [14], were obtained from the Liver Cancer Institute of Zhongshan Hospital, Fudan University, Shanghai, China. One normal human liver cell line L02 [15]and one mouse fibroblast cell line NIH3T3[16] was obtained from the Central Laboratory of Shandong Provincial Hospital. HCC cells were routinely cultured in Dulbecco's modified Eagle's medium (DMEM) with high glucose (Hyclone, USA). L02 and NIH3T3 cells were cultured in RPMI 1640 medium (Hyclone, USA). Both the DMEM and the RPMI 1640 medium were supplemented with 10% fetal bovine serum (FBS, Gibco, USA), antibiotics (100 U/ml penicillin, 2 μg/ml streptomycin) and 2 mmol/L glutamine, at 37°C in a humidified, 5% CO_2 _atmosphere.

### Immunocytochemistry

MHCC-97H, MHCC-97L and Hep3B cells were cultured in 24-well plates with one glass slide in each well. Twenty four hours later, the slides were washed with PBS, fixed with 4% paraformaldehyde for 30 min and permeablized with 0.2% Triton X-100 for 20 minutes. In order to inhibit the endogenous peroxidase activity, the slides were treated with 3% H_2_O_2 _for 15 min. The nonspecific binding sites were blocked by incubation in a solution of 5% bovine serum albumin (BSA) for 20 min. The primary rabbit polyclonal antibody to PDCD4 (Santa Cruz Biotechnology, Santa Cruz, California, USA. diluted by 1:30 in phosphate-buffered saline, PBS) was applied and incubated at 4°C, overnight. Slides were washed twice with PBS and incubated with biotinylated goat anti-rabbit IgG (Santa Cruz Biotechnology, Santa Cruz, California, USA) at a 1:100 dilution. Slides were then incubated for 30 min with HRP-conjugated streptavidin (Zhongshan Biotechnology, Beijing, China). The avidin/biotin complexes were revealed with a diaminobenzidine (DAB) kit (Zhongshan Biotechnology, Beijing, China) according to the manufacturer's instructions. Hematoxylin was used to counterstain the slides which were then dehydrated and cover-slipped. Equal volume of PBS was used instead of the primary antibody and served as a negative control [17].

A semi-quantitative scoring method was used to assess the expression level of PDCD4. The histological score (HSCORE) was calculated with the following equation: HSCORE = ΣPi (i+1), where i = intensity of staining with a value of 1, 2, 3 or 4 (weak, moderate, strong or very strong, respectively) and Pi is the percentage of stained cells for each intensity grade, varying from 0 to 100%. In each slide, five different areas were evaluated under a microscope with 200-fold original magnification, the percentage of the cells for each intensity grade within these areas was determined by two investigators at different times, and the average score was used[18].

### RNA isolation and real-time PCR

Total RNAs of MHCC-97H, MHCC-97L or Hep3B cells were extracted by Trizol (Invitrogen) reagent and 0.5 μg of each kind of RNA was reversely transcripted into first-strand cDNA with the RT reagent kit (Takara, Dalian, China) according to the manufacturer's protocol. Real-time quantitative PCR was performed with a QuantiTect SYBR Green kit (TaKaRa, Dalian, China) in a 10 μl reaction volume, which contained 5 μl of SYBR^® ^Green I PCR mix, 0.2 μM of forward and reverse primer, 1 μl of diluted cDNA template, and appropriate amounts of sterile ddH_2_O. Conditions for PCR of the other molecules were as follows: 5 min at 95°C; 40 cycles of 15 s at 95°C and 60 s at 60°C; 15 s at 95°C and 15 s at 60°C. The entire experiments were repeated at least three times. All quantifications were performed with human glyceraldehyde-3-phosphate dehydrogenase (GAPDH) as an internal standard. Primer sequences used in the PCR were as follows: PDCD4: 5'-CAGTTGGTGGGCCAGTTTATTG-3' (sense), 5'-AGAAGCACGGTAGCCTTATCCA-3' (antisense); MTA1: 5'-AAGCACGCAACCCTGTCAGTC-3' (sense), 5'-TCTCGGGCAGGTCCACCATTT-3' (antisense); GAPDH: 5'-ACAGCGACACCCACTCCTCC-3' (sense), 5'-TAGCCAAATTCGTTGTCATACCAG-3' (antisense). Real-time PCR was carried out on an ABI PRISM 7500 Sequence Detection System (Applied Biosystems, NJ, USA), and results were analyzed using the integrated Sequence Detection System Software Version 1.4. The relative quantification (RQ) of gene expression was analyzed by the 2^-ΔΔCt ^method and the results were expressed as extent of change with respect to control values [19].

### Plasmid construction

RNA was isolated from the L02 cells using Trizol reagent (Invitrogen). The RT reagent kit (Jingmei Biotech, Shenzhen, China) was used to transcript RNA into cDNA according to the manufacturer's instructions. The whole coding sequence of human PDCD4 gene (Genbank accession no. [BC026104.2]) was amplified by polymerase chain reaction (PCR) with primers: 5'-CTCTAGAATGGATGTAGAAAATGAGCAG-3' (154–174) (sense), and 5'-GCGGTACCTCAGTAGCTCTCTGGTTTAAG-3' (1563-1543) (antisense). The XbaI and EcoRI restriction sites were introduced to the primers, respectively. The final volume of reaction was 80 μl, containing 1 μl (≤ 1 μg) of cDNA mixture, 10 × PCR buffer 8 μl, 1.0 μl of each dNTP, 0.5 μl of Taq polymerase, 1.0 μl of each PDCD4 gene primer. The PCR amplification was performed for 35 cycles as follows: at 95°C for 2 min, at 90°C for 30 s, at 56°C for 30 s, and at 72°C for 90 s, with a final extension at 72°C for 10 min. The PCR product was digested by KpnI and subcloned into EcoRI and XbaI sites of the pcDNA3.1 (-) vector (Invitrogen) to generate pcDNA3.1 (-)-PDCD4 construct. The recombinant was identified by double digestion with restriction enzymes and DNA sequencing was performed by Shanghai Sangon Biological Engineering Technology and Service Co. Ltd (Sangon).

### Cell transfection

In order to study the effects of PDCD4, we transfected the pcDNA3.1 (-)-PDCD4 plasmid into MHCC-97H cells which was shown to express lowest level of PDCD4. Cells were grown to 70% confluence in 6-well plates, pcDNA3.1 (-)-PDCD4 plasmid (2 μg per well) was transfected by LipofectAMINE 2000 (Invitrogen) according to the manufacturer's instructions. The empty vector pcDNA3.1 (-) was also transfected as a control. The parental cells without transfection were considered to be another control group. Stable clones were generated by selection in complete culture medium containing 400 μg/mL G418 48 h later. Western blot analysis was taken to identify the effectiveness of the PDCD4 transfection[20].

### Western blot analysis

Western blot analyses were performed to detect the expression of PDCD4, to identify the effectiveness of the PDCD4 gene transfection and to analyze the expression of MTA1 after PDCD4 transfection. HCC cells grown to 70–90% confluence were washed with ice-cold PBS for two times and then collected by scraping. The cell pellets were homogenized in extraction buffer (50 mM Tris-HCl, 0.1% SDS, 150 mM NaCl, 100 μg/ml phenylmethylsulfonyl fluoride, 1 μg/ml aprotinin, 1% Nonidet P-40, and 0.5% sodium orthovanadate), then incubated at 4°C for 30 min and centrifuged 20 min at 12,000 g/min. The total proteins (50 μg per lane) were resolved in 10% SDS-polycrylamide gels, and then transferred onto nitrocellulose membrane (0.45 μm, Millipore, Bedford, MA, USA) in 25 mM Tris-base, 190 mM glycine, and 20% methanol using a semi-dry blotter. Following blocking with 10% nonfat milk and 0.1% Tween20 in TBS for 2 h, the membranes were incubated with anti-PDCD4(Santa Cruz Biotechnology, Santa Cruz, California, USA, 1:200 for gene expression and 1:2000 for identification of transfection), anti-MTA1(Santa Cruz Biotechnology, Santa Cruz, California, USA, 1:500) or anti-β-actin (Jingmei Biotech, Shenzhen, China, 1:2000), respectively, at 4°C overnight. After binding of horseradish peroxidase (HRP)-coupled goat anti-mouse or goat anti-rabbit IgG (Jingmei Biotech, Shenzhen, China,1:5000) at room temperature for 2 h, antigens were visualized by enhanced chemiluminescence (Santa Cruz Biotechnology, Santa Cruz, California, USA,) Bands corresponding to different proteins were scanned and the respective areas and IOD were determined using Image-Pro Plus 6.0. The relative densities were calculated by normalizing the IOD of each blot with that of β-actin [21].

### MTT assay for cell proliferation

Cell proliferation rates of MHCC-97H-PDCD4, MHCC-97H-vector and MHCC-97H cells were evaluated with 3-(4, 5-Dimethylthiazol-2-yl)-2, 5-diphenyl-tetrazolium bromide (MTT) assay. An equal number of cells (5 × 10^3^) from the different stable cell lines of MHCC-97H-PDCD4 (Group 1), MHCC-97H-vector (Group 2) and MHCC-97H (Group 3) were seeded in triplicate with serum-containing medium in six 96-well plates. At 0–5 day of culture, MTT assay was performed daily using one plate. The medium was replaced with 100 μl of fresh serum-free medium containing 20 μl each time. The cells were incubated at 37°C for an additional 4 h. After the removal of the medium, 100 μl of dimethyl sulfoxide (DMSO) was added, and the formation of colored formazan dye was assessed at 490 nm. The experiment was was repeated 3 times [22].

### Cell cycle analysis

The cell cycle distribution of MHCC-97H cells was assessed based on their DNA contents and detected by the DNA Reagent Kit (Beckman Coulter, Fullerton, California, USA), according to the manufacturer's protocol. Twenty-four hours after transient transfection, MHCC-97H cells were trypsinized, washed with PBS, suspended in 100 μl PBS and fixed with 70% alcohol for 30 minutes on ice. Cells were then washed with cold PBS twice and resuspended in hypotonic solution [0.1% sodium citrate, 0.2% Nonidet P-40 (NP-40)] and then incubated with 50 μg/mL propidium iodide and 0.25 mg/mL RNase A at 4°C for 30 min in the dark. After incubation at 37°C for further 15 min, the DNA contents were analyzed on a flow cytometry (Beckman-Coulter, Fullerton, California, USA) [23]. According to the DNA contents, the percentage of G1, S and G2 were determined. PI was then calculated as follows: PI = (S+G_2_)/(S+G_2_+G_1_) [24].

### Flow cytometric assay for cell apoptosis

Flow cytometry was used to evaluate cell apoptosis 24 hours after transient transfection. According to the manufacturer's instructions, the MHCC-97H cells undergoing apoptosis were determined by the Annexin V-FITC/PI apoptosis assay kit (Jingmei Biotech, Shenzhen, China). The cells were trypsinized, washed with PBS, suspended in 100 μl PBS and fixed with 70% alcohol for 30 minutes on ice. Cells were then washed with cold PBS twice, resuspended in ice-cold binding buffer and incubated with Annexin V-FITC and PI for 10 min prior to flow cytometry analysis[25].

### Hoechst 33258 staining for apoptotic morphology

Hoechst 33258 staining was performed 24 h after transit transfection. MHCC-97H cells were stained with Hoechst 33258 (5 μg/ml, Sigma) for 10 min at room temperature in the dark, washed three times with PBS and analyzed with a fluorescence microscope. At least 200 cells were counted and the percentage of apoptotic cells were calculated[26].

### Migration and Matrigel invasion assay

Cell migration and invasion tests were performed in Transwell chambers (Corning Coster; Cambridge, MA) equipped with a filter membrane with 8-μm pores, coated with(for invasion assay) or without(for migration assay) 50 μg Matrigel (Sigma). Fibroblast conditioned medium (NIH3T3-CM), containing a mixture of molecules and capable of stimulating the migration of invasive cells, was used as chemoattractant in these experiments [27]. MHCC-97H-PDCD4, MHCC-97H-vector or MHCC-97H cells were suspended in 100 μl DMEM containing 10% FBS and plated at 1 × 10^5 ^cells/well onto the upper compartment of the chamber. The lower chambers were filled with 600 μl NIH3T3-CM, which was obtained by a 24 h incubation of NIH3T3 cells with 50 μg/ml ascorbic acid in serum-free DEME media [16]. Cells were cultured at 37°C in a humidified, 5% CO_2 _atmosphere for another 24 hours. The filters were then washed with PBS, fixed with 95% methanol for 20 min and stained with hematoxylin and eosin solution. Cells on the upper surface of the filters were gently removed with cotton swabs. The number of cells that had migrated to the lower surface of the filter membrane was counted in five randomly chosen fields under a light microscope (× 200). The average number of migrated cells per microscopic field was analyzed[28].

### Statistical Analyses

Data were reported as means ± SD of the combined experiments. Student's two-tailed t test for independent means was employed to determine significant differences (P < 0.05). Analyses were performed using SPSS16.0 statistical program.

## Results

### Expression of PDCD4

**Figure 1:**
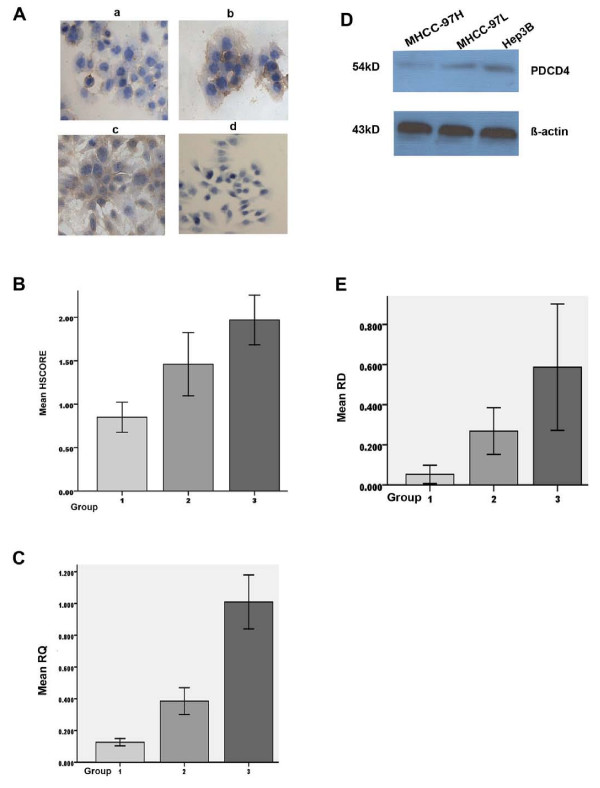
**Expression of PDCD4 in HCC cells**. A: Immunocytochemical staining. The positive staining(×200) was brownish and localized in cytoplasm. D: Western blot assay. Representative figures are shown from one of three individual experiments. B, C or E shows statistical analysis for immunocytochemical staining, real – time PCR or western blot assay, respectively. In A, a, b or c represents cells of MHCC-97H, MHCC-97L or Hep3B, respectively; d shows cell staining without the primary antibody. In B, C and E, Group1, Group 2 or Group3 represents cells of MHCC-97H, MHCC-97L and Hep3B, respectively. Bars represent the means ± SD. The difference between Group1 and Group2 (P < 0.05) or Group1 and Group3 (P < 0.01 in B; P < 0.05 in E) was significant.

The expression of PDCD4 in three different metastatic potential HCC cell lines was detected. The positive immunocytochemical staining for PDCD4 was brownish and localized in cytoplasm (Fig. 1A). HSCORE for MHCC-97H cells, MHCC-97L cells and Hep3B cells was 0.85 ± 0.17, 1.46 ± 0.36 and 1.97 ± 0.29, respectively (Fig. 1B). Difference between Group1 and Group2 (n = 5, P < 0.05) or Group1 and Group3 (n = 5, P < 0.01) or Group2 and Group3 (n = 5, P < 0.05) was significant.

The quantitative assay by real time PCR was reported in RQ units as compared with the noninvasive Hep3B cells (Fig. 1C). RQ for MHCC-97H cells and MHCC-97L cells was 0.126 ± 0.023 and 0.385 ± 0.084, respectively. The mean RQ for Group1 and Group2 was 0.126 ± 0.023 and 0.385 ± 0.084, respectively. The difference between Group1 and Group2 was significant (n = 3, P < 0.05).

Western blots for PDCD4 expression display a band of 54 kD (Fig. 1D). The relative densities (RD) of PDCD4 for Group1, Group2 and Group3 were 0.053 ± 0.045, 0.268 ± 0.067 and 0.587 ± 0.182, respectively (Fig. 1E). Difference between Group1 and Group2 or Group1 and Group3 was significant (n = 3, P < 0.05). There is no difference between Group2 and Group3 (n = 3, P > 0.05).

Data of the above experiments showed that the highest metastatic potential MHCC-97H cells expressed lowest level of PDCD4. The expression level of PDCD4 was inversely correlated with the metastasis potentials of HCC cells.

### Plasmid construction and efficiency of PDCD4 transfection

**Figure 2:**
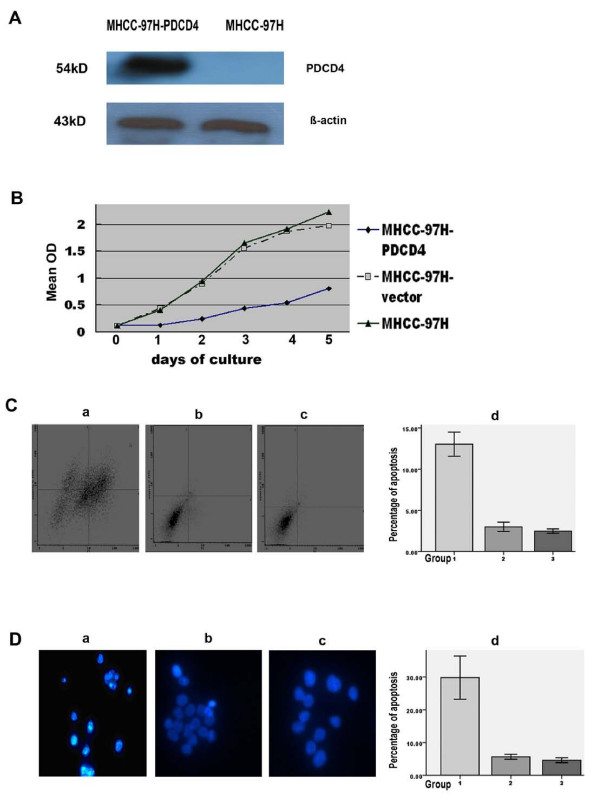
**Effects of PDCD4 on MHCC-97H cell proliferation and apoptosis**. A: Western blot analysis for identification of transfection efficiency. B: MTT assay for cell proliferation. C: Flow cytometric assay for cell apoptosis. D: Hoechst 33258 staining for cell apoptosis (×200). Morphological changes of cell apoptosis were shown as chromatin condensation and nuclear fragmentation. Representative images are shown from three individual experiments. In C and D, a or Group1, b or Group 2, and c or Group3 represents cells of MHCC-97H-PDCD4, MHCC-97H-vector and MHCC-97H, respectively; d shows statistical analysis for each assay. Bars represent the means ± SD. The difference between Group1 and Group2 or Group3 was significant (P < 0.01).

A plasmid pcDNA3.1 (-)-PDCD4 encoding the PDCD4 gene was constructed. The recombinant was identified by double digestion with restriction enzymes and sequencing analysis. DNA sequencing of the recombinant pcDNA3.1 (-)-PDCD4 was also identified by Sangon. The efficiency of PDCD4 gene transfection was identified by western blot analysis (Fig. 2A).

### Effects of PDCD4 on MHCC-97H cells proliferation

The MHCC-97H cell proliferation rate was assayed by MTT. The detected absorbance at 490 nm of the MHCC-97H-PDCD4 group was 0.543 ± 0.150, which was lower than that of the MHCC-97H-vector group (1.343 ± 0.268) or MHCC-97H group (1.278 ± 0.258). The difference was significant (n = 3, P < 0.05). No statistical difference was found between the two control groups (n = 3, P > 0.05) (Fig. 2B).

To further testify the effect of PDCD4 on proliferation of HCC cells, cell cycle analysis with a flow cytometer was performed and the proliferative indexes (PI) were calculated. As shown in Table 1, an increase of percentage both in G1 stage and in G2 stage was observed in MHCC-97H-PDCD4 cells, accompanied by a corresponding reduction in the percentage of cells in S phase. PI was 27.83 ± 0.95%, 42.47 ± 2.90% and 44.47 ± 2.37% for the MHCC-97H-PDCD4 cells, the MHCC-97H-vector and the MHCC-97H cells, respectively. The difference of G1, G2, or S percentage and PI between the MHCC-97H-PDCD4 cells and the MHCC-97H-vector or the MHCC-97H cells is significant (n = 3, P < 0.05). No significant difference was found between the MHCC-97H-vector and the MHCC-97H cells. These data indicate that PDCD4 might promote both G_1 _and G2 arrest in MHCC-97H cells and further block the proliferation of HCC cells.

**Table 1:** Data of effect of PDCD4 on MHCC-97H cell cycle distribution

**Cell line**	**phase of cell**			**PI**
	**G1**	**S**	**G2**	

MHCC-97H-PDCD4	70.83 ± 3.53	23.50 ± 0.20	5.50 ± 0.58	29.05 ± 0.28

MHCC-97H-vector	67.33 ± 1.02	31.13 ± 0.44	1.90 ± 0.45	32.98 ± 0.89

MHCC-97H	67.43 ± 0.75	30.63 ± 0.98	1.93 ± 0.47	32.57 ± 0.75

The cell-cycle distribution was assessed by flow cytometric analysis 24 h after

transfection of PDCD4 to MHCC-97H cells. The data shown are means ± SEM of percentage of G1, G2 or S phase in three experiments. The proliferative indexes (PI) were calculated as follows: PI = (S+G_2_)/(S+G_2_+G_1_). The difference of PI between the MHCC-97H-PDCD4 group and MHCC-97H-vector or the MHCC-97H group is significant (n = 3, P < 0.05). No significant difference between the MHCC-97H-vector and the MHCC-97H group is found.

### Effects of PDCD4 on MHCC-97H cell apoptosis

Cell apoptosis was analyzed both quantitatively and morphologically. The apoptosis rate detected by the flow cytometric assay was 13.03 ± 1.47%, 2.99 ± 0.33% and 2.47 ± 0.15% in the MHCC-97H -PDCD4 cells (Group1), the MHCC-97H-vector cells (Group2) and the MHCC-97H cells (Group3), respectively (Fig. 2C).

Hoechst 33258 staining showed the nuclear alterations of apoptosis – condensed, coalesced, and segmented nuclei with a brighter blue fluorescence. The percentage of apoptosis cells was 29.84 ± 3.80% in MHCC-97H -PDCD4 group(Group1), 5.666 ± 0.44% in the MHCC-97H-vector group (Group2) and 4.62 ± 0.43% in the MHCC-97H group (Group3), respectively. (Fig. 2D). The difference was significant between Group1 and Group2 or Group3 (n = 5, P < 0.01). There was no statistical difference between the two control groups.

### Effects of PDCD4 on MTA1 expression of MHCC-97H cells

**Figure 3:**
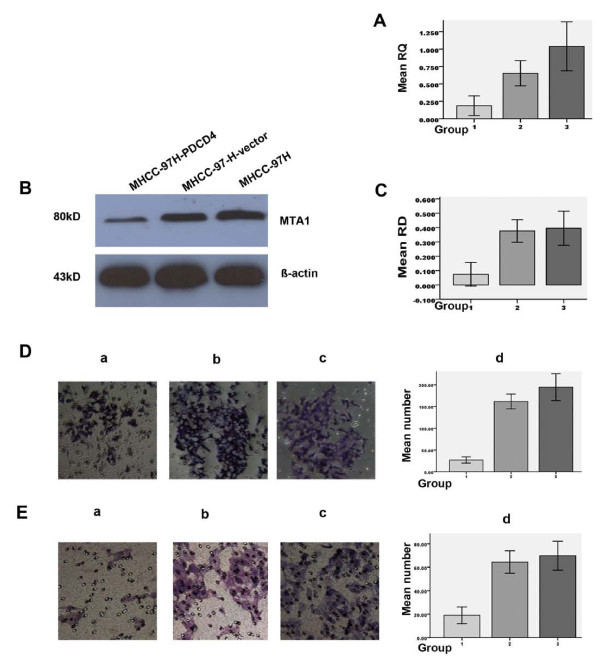
**Effects of PDCD4 on MHCC-97H cell metastatic potential**. B: Western blots for MTA1 expression. A and C: Statistical analysis for MTA1 expression with real-time PCR and western blot assay, respectively. D: Cell migration assay. E: Matrigel invasion assay. Representative images are shown from three individual experiments. In A, C, D and E, a or Group1, b or Group 2, and c or Group3 represents cells of MHCC-97H-PDCD4, MHCC-97H-vector and MHCC-97H, respectively. Bars represent the means ± SD. Difference between Group1 and Group2 or Group1 and Group3 was significant (P < 0.05 in A and C; P < 0.01 in D and E).

In order to further study the effects of PDCD4 on metastasis, we detected the gene expression of MTA1 in MHCC-97H-PDCD4, MHCC-97H-vector and MHCC-97H cells, respectively, with both real- time PCR and western blotting analysis. The quantitative assay of real- time PCR was reported in RQ units as compared with the parental MHCC-97H cells. RQ for the recombinant group and the empty vector group was 0.187 ± 0.083 and 0.652 ± 0.105, respectively. The difference was significant (n = 3, P < 0.05) (Fig. 3A). Western blots for PDCD4 expression display a band of 80 kD (Fig. 3B). The relative densities (RD) of MTA1 for MHCC-97H cells, MHCC-97L cells and Hep3B cells were 0.074 ± 0.047, 0.376 ± 0.045 and 0.395 ± 0.069, respectively (Fig. 3C). The difference was significant (n = 3, P < 0.05).

### Effects of PDCD4 on MHCC-97H cell migration and invasion

In the migration assay, the average number of migrated cells per field of the MHCC-97H -PDCD4 group (Group1) was 27.20 ± 7.26, which was much lower than that of the MHCC-97H -vector group (Group2) (161.80 ± 17.06) or the MHCC-97H group (Group3) (194.60 ± 30.83) (Fig. 3D). The average number of migrated cells in the invasion assay was 19.0 ± 3.18, 64.40 ± 9.61 and 69.80 ± 12.32 for the Group1, Group2 and Group3, respectively (Fig. 3E). The difference was significant between Group1 and Group2 or Group3 (n = 5, P < 0.01). There is no difference between Group2 and Group3.

## Discussion

PDCD4 was originally found to be an apoptosis-associated gene in mouse cells. PDCD4 expression was found to be up-regulated in cells treated with various apoptosis-inducing agents such as topoisomerase inhibitors, corticosteroids and cytokine deprivation[29]. The function of PDCD4 in the course of programmed cell death remains unclear. Later studies showed that PDCD4 was a suppressor of tumor cell transformation. The expression levels of PDCD4 were reduced in many human progressed carcinomas[7]. A study on human HCC showed that expression level of PDCD4 protein was much lower in HCC tissues tested than that of the corresponding noncancerous liver[30].

In this study, we showed that higher metastatic potential HCC cells expressed lower level of PDCD4. The expression levels of PDCD4 were inversely correlated with the metastasis potentials of HCC cells. This result is consistent with the previous findings. We also demonstrated that the MHCC-97H cell proliferation rate was remarkably decreased and the cell apoptosis rate was significantly increased after transfection with the PDCD4 gene. Cell cycle analysis showed that transfection of PDCD4 gene increase the percentage of both G1 and G2. Data of our results suggest that PDCD4 might promote cell cycle arrest in phase of G1 and in G2 and further block the cell proliferation. It is known that PDCD4 is a binding partner of the eukaryotic translation initiation factor 4A (eIF4A). By binding to eIF4A, PDCD4 can directly inhibit translation initiation and then delay the process of protein synthesis. A study on Bon-1 carcinoid cells showed that PDCD4 not only suppressed the transcription of the mitosis-promoting factor cyclin-dependent kinase 1(CDK1)/cdc2, but also decreased the expression of CDK4/6[31]. CDK1 and CDK4/6 are are directly involved in cell cycle control. Decrease of CDK1 or CDK4/6 promotes cell cycle arrest in G1 or G2 phase and further inhibits proliferation of cells[32].

PDCD4 inhibits the activity of c-Jun N-terminal kinase (JNK), blocks the JNK signaling pathway and consequently decreases the activation of c-Jun and AP-1-dependent transcription[8]. Many genes regulated by AP-1 are important modulators of invasion and metastasis. Decrease of expression or inhibition of activation of AP-1 results in inhibition of tumor cell motility and invasiveness[33].

Our study revealed that the expression of the MTA1gene was remarkably decreased after the PDCD4 gene transfection. In the migration and Matrigel invasion assay, we discovered that the MHCC-97H cells migrated to the lower surface were greatly decreased after PDCD4 gene transfection. A study on a human acute myeloid leukemia (AML) cell line NB4 demonstrated that Knockdown of PDCD4 by RNA interference (siRNA) leads to induction of c-myc, suggesting that c-myc maybe a potential down-stream target of PDCD4[7]. MTA1 is an integral subunit of nucleosome remodeling and histone deacetylation (NuRD) complex which contains both histone deacetylase and nucleosome remodeling activity. It has been shown to be overexpressed in metastatic carcinomas. Recent studies on rat fibroblasts cells revealed that MTA1 is one of the essential first down-stream effectors of the c-myc oncoprotein. Activation of c-myc causes induction of the MTA1 expression [34]. In MHCC-97H cells stably transfected with the PDCD4, activity of c-myc maybe inhibited and the gene expression of MTA1 is further blocked.

Metastasis is a multistep process. Cell migration and invasion are essential for tumor progression and metastasis. Matrigel is a reconstituted basal membrane with most components of extracellular membrane. Malignant cells have to degrade the surrounding ECM before spread [35]. Metastatic potential of MHCC-97H cells had been found to be correlated to the number of cells migrated in the migration and invasion assay [14].

In summary, we showed that the expression of PDCD4 was inversely correlated to the metastatic potentials of HCC cells. PDCD4 effectively blocked the proliferation rate, decreased the gene expression of metastasis associated protein1, and inhibited the migration and invasion activities of MHCC-97H cells. These results demonstrate that PDCD4 might be a novel suppressor to metastatic potential of HCC cells. By our knowledge, this was the first observation to investigate the effects of PDCD4 on metastatic potential of HCC cells. Further studies are required to confirm these findings in vivo.

## Abbreviations

HCC: Hepatocellular carcinoma; PDCD4: Programmed cell death 4; MHCC-97H: high metastatic potential; MHCC-97L: low metastatic potential; MTA1: metastasis tumor antigen 1; DMEM: Dulbecco's modified Eagle's medium; FBS: fetal bovine serum; PBS: phosphate-buffered saline; BSA: bovine serum albumin; DAB: Diaminobenzidine; HSCORE: histological score; GAPDH: glyceraldehyde-3-phosphate dehydrogenase; PCR: polymerase chain reaction; HRP: Horseradish peroxidase; MTT: 3-(4, 5-Dimethylthiazol-2-yl)-2, 5-diphenyl-tetrazolium bromide; DMSO: dimethyl sulfoxide; JNK: c-Jun N-terminal kinase; AML: acute myeloid leukemia; siRNA: RNA interference; NuRD: nucleosome remodeling and histone deacetylation.

## Competing interests

The authors declare that they have no competing interests.

## Authors' contributions

SZ carried out most parts of the experiment; JL, YJ and YX participated in the experiment; CQ participated in the design of the study.
